# Supplementary material for: Impact of interannual and multidecadal trends on methane-climate feedbacks and sensitivity
Source: Nat Commun. 2022 Jun 23;13:3592. doi: 10.1038/s41467-022-31345-w (PMC9226131; doi:10.1038/s41467-022-31345-w)
Supplement: Supplementary file 1 — Supplementary information [file 41467_2022_31345_MOESM1_ESM.pdf]

Impact of Interannual and Multidecadal Trends on Methane-Climate Feedbacks and Sensitivity

Supplementary Information

Chin-Hsien Cheng[1,2], Simon A. T. Redfern[2,3*]

[1]Joint International Research Laboratory of Climate and Environment Change, Nanjing University of Information Science and Technology (NUIST), Nanjing 210044, China

[2]Asian School of the Environment, Nanyang Technological University, 50 Nanyang Avenue, Singapore 639798

[3]School of Materials Science and Engineering, Nanyang Technological University, 50 Nanyang Avenue, Singapore 639798

Correspondence to: Simon A. T. Redfern (simon.redfern@ntu.edu.sg)

Supplementary Table 1. The mean global observed $\Delta C_{CH4}/\Delta t$ (ppb yr^{-1}) and estimates of *T&Pr* contributions during five characteristic periods with different assumptions (exclusive land-mean, sea-mean, and area-weighted-mean).

	$\Delta C_{CH4}/\Delta t$	exclusive sea-contributions		exclusive land-contributions		area-mean-contributions		*nc*-contributions (Obs. - Max Est.)	
Periods	Obs.	Est.	σ_{SST}	Est.	$\sigma_{LSAT+Pr}$	Est.	$\sigma_{T\&Pr}$	Est.	$1-\sigma_{T\&Pr}$
7/1985-6/1992	11.04	5.70	52%	5.36	49%	5.98	54%	5.06	46%
7/1992-12/1998	5.49	4.08	74%	3.96	72%	4.27	78%	1.22	22%
1/1999-12/2005	0.64	0.55	86%	0.21	32%	0.42	66%	0.09	14%
1/2006-12/2011	5.18	2.52	49%	3.03	59%	2.98	58%	2.15	41%
1/2012-12/2018	8.00	5.60	70%	5.98	75%	5.99	75%	2.01	25%

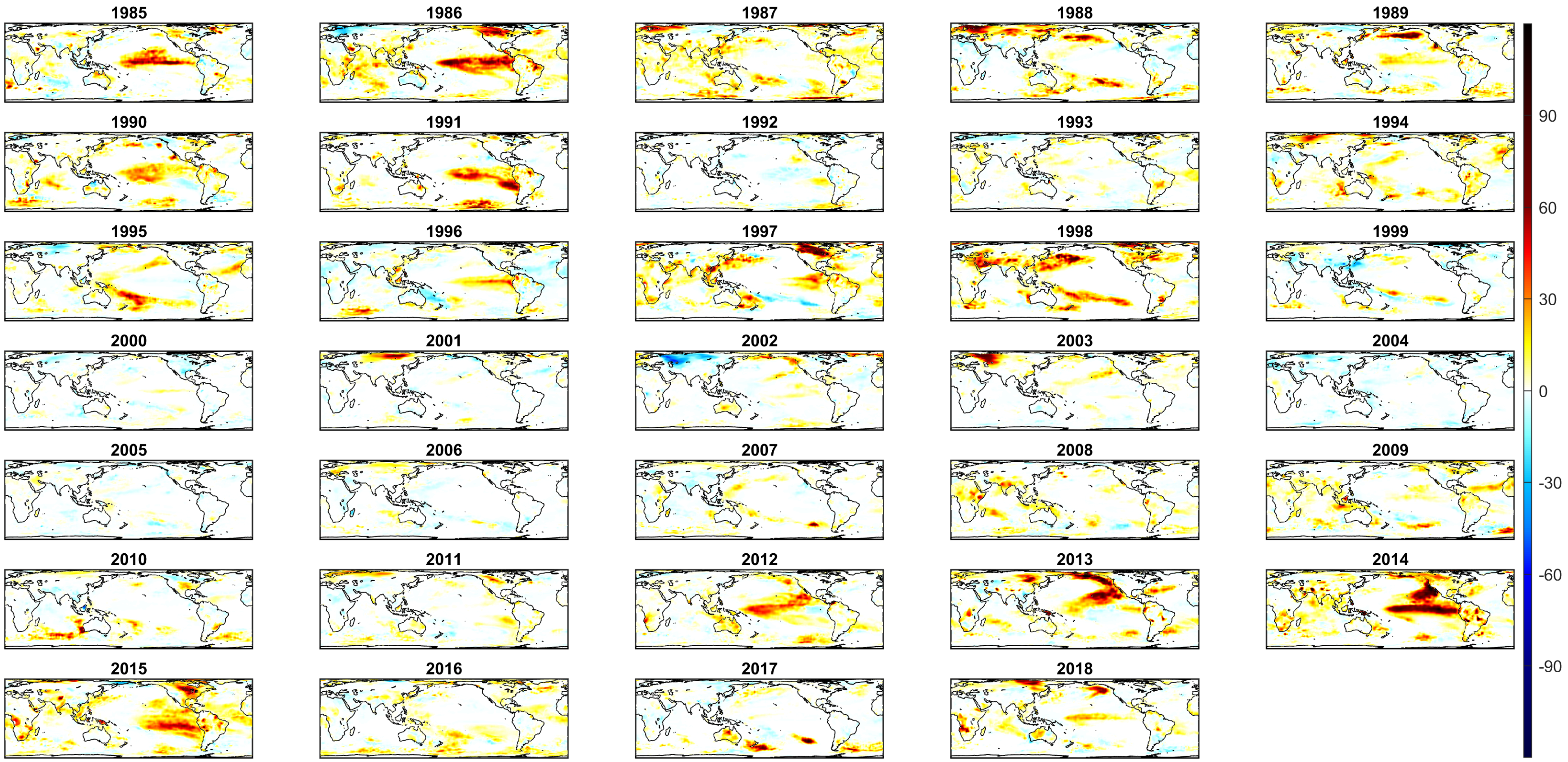

Supplementary Fig. 1. Yearly $\partial C_{CH4,T\&Pr}/\partial t$ maps with positive *T*-correlation (positive feedback) and negative *Pr*-correlation. Note that *SST* significantly contribute positively to the increasing C_{CH4} before 1997 and after 2012, but not during 1998-2011. This is consistent with the multidecadal warm-cool variability.

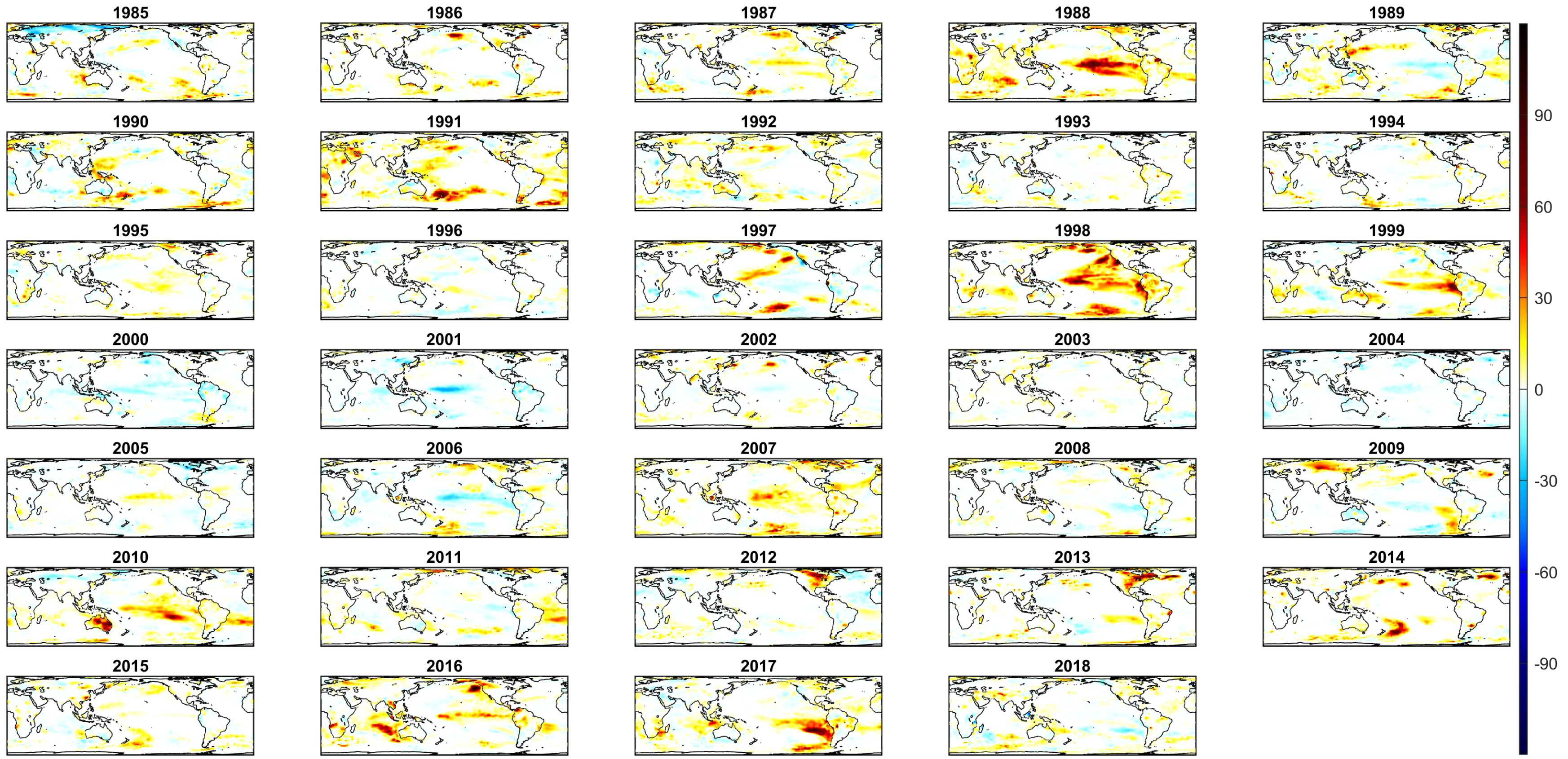

Supplementary Fig. 2. Yearly $\partial \boldsymbol{C}_{CH4,T\&Pr}/\partial \boldsymbol{t}$ maps with negative *T*-correlation (negative feedback) and positive *Pr*-correlation. Note that *SST* positively contributes to the increasing $\boldsymbol{C}_{CH4}$ mainly occurs during the cooling years after El Niño (e.g. 1988 after 1986-1987 El Niño, 1998-1999 after 1997-1998 El Niño, and 2016-2017 after 2015-2016 El Niño. This marks the interannually lagged responses of positive feedback via negative feedback process with weakened *SST*-H_2O-OH sink.

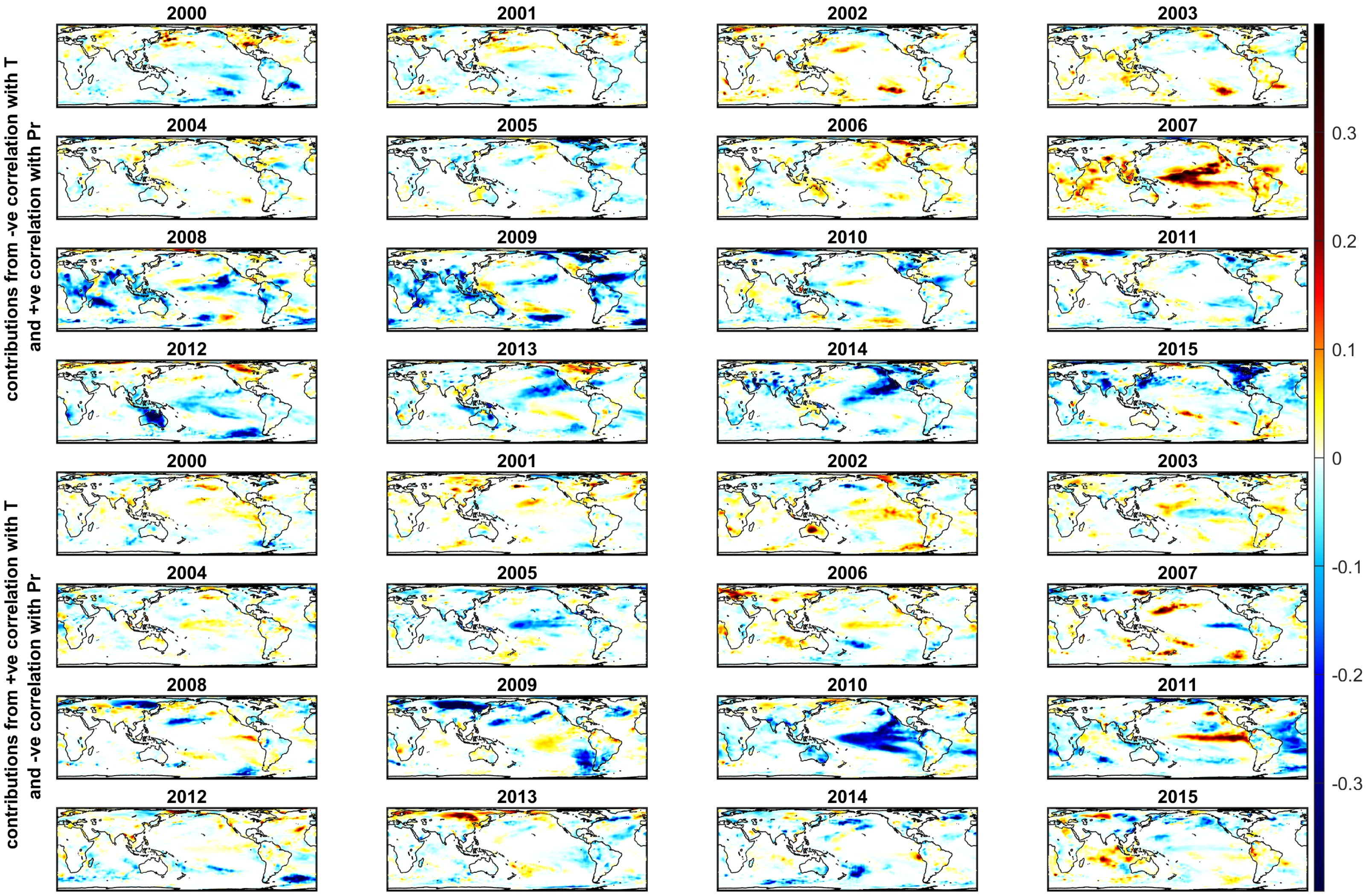

Supplementary Fig. 3. Yearly $\partial(\delta^{13}CH_4)_{T\&Pr}/\partial t$ maps with different correlation signs. Upper: negative *T*-correlation (positive feedback) and positive *Pr*-correlation. Lower: postive *T*-correlation (negative feedback) and negative *Pr*-correlation.

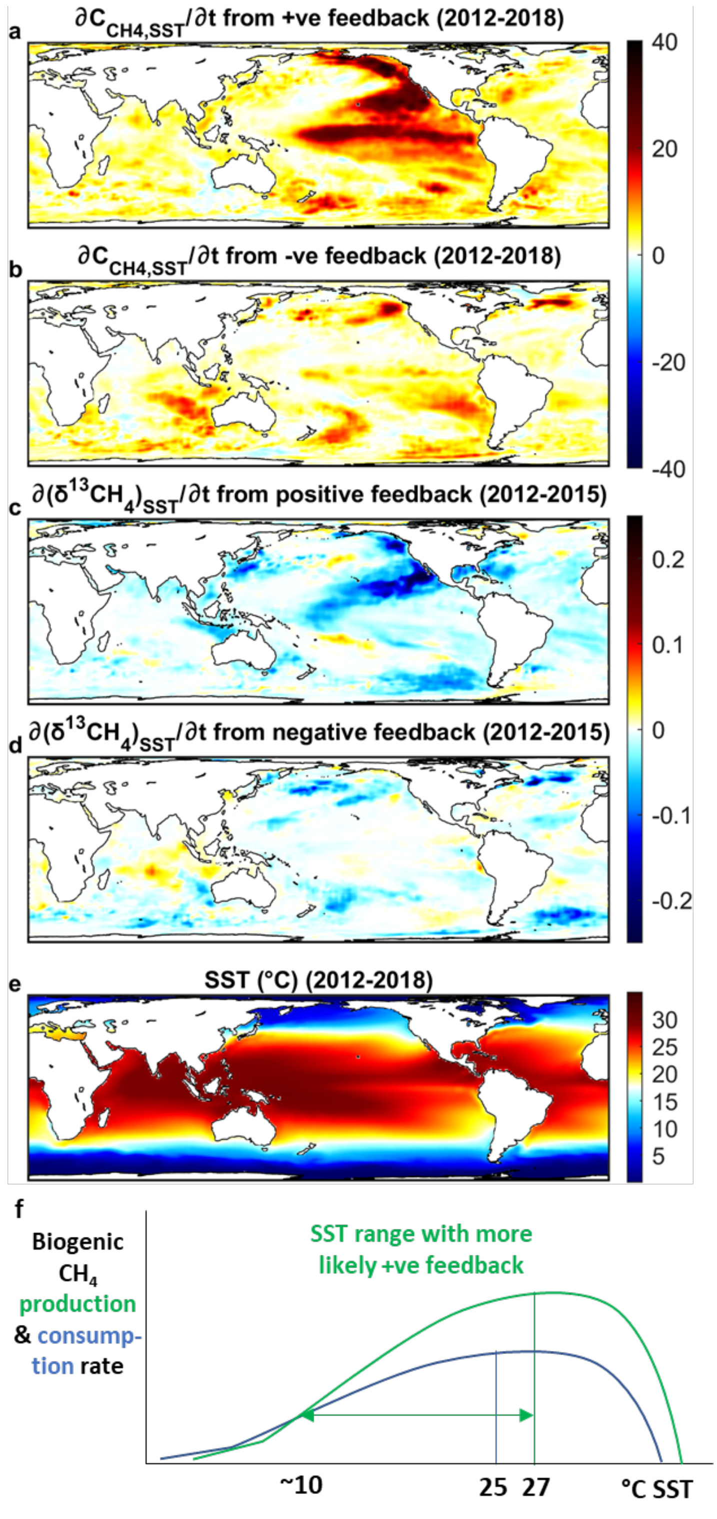

Supplementary Fig. 4. Comparison of *c*-contributions from positive (a,c) and negative (b,d) *SST*-feedbacks since 2012, the mean SST (e), and conceptual illustration of a possible reason for this net positive SST-feedbacks (f).

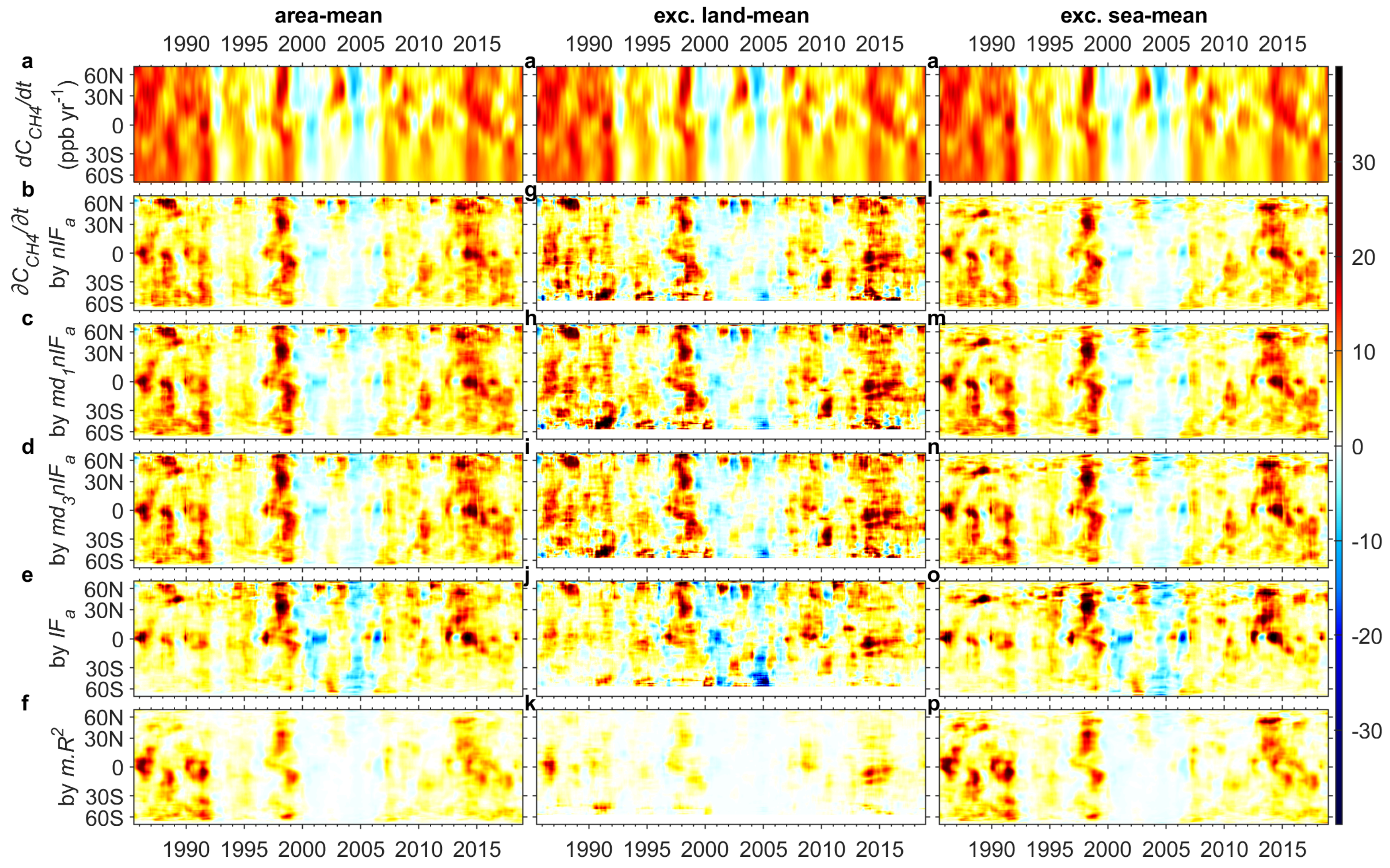

Supplementary Fig. 5. Comparison between the observed $dC_{CH4}(T\&Pr)/dt$ (1st row) and estimated $\partial C_{CH4}(T\&Pr)/\partial t$ *c*-contributions given by various methods. The estimates given by *nIF* are based on the original *nIF* proposed by Liang (equation M6). The estimates given by the 3rd modified *nIF* (md_3nIF) are based on equation M7, and the estimates given by the 1st modified *nIF* (md_1nIF) are based on normalizer $Z = |IF(X \rightarrow Y)| + |IF(\text{non}X \rightarrow Y)|$.

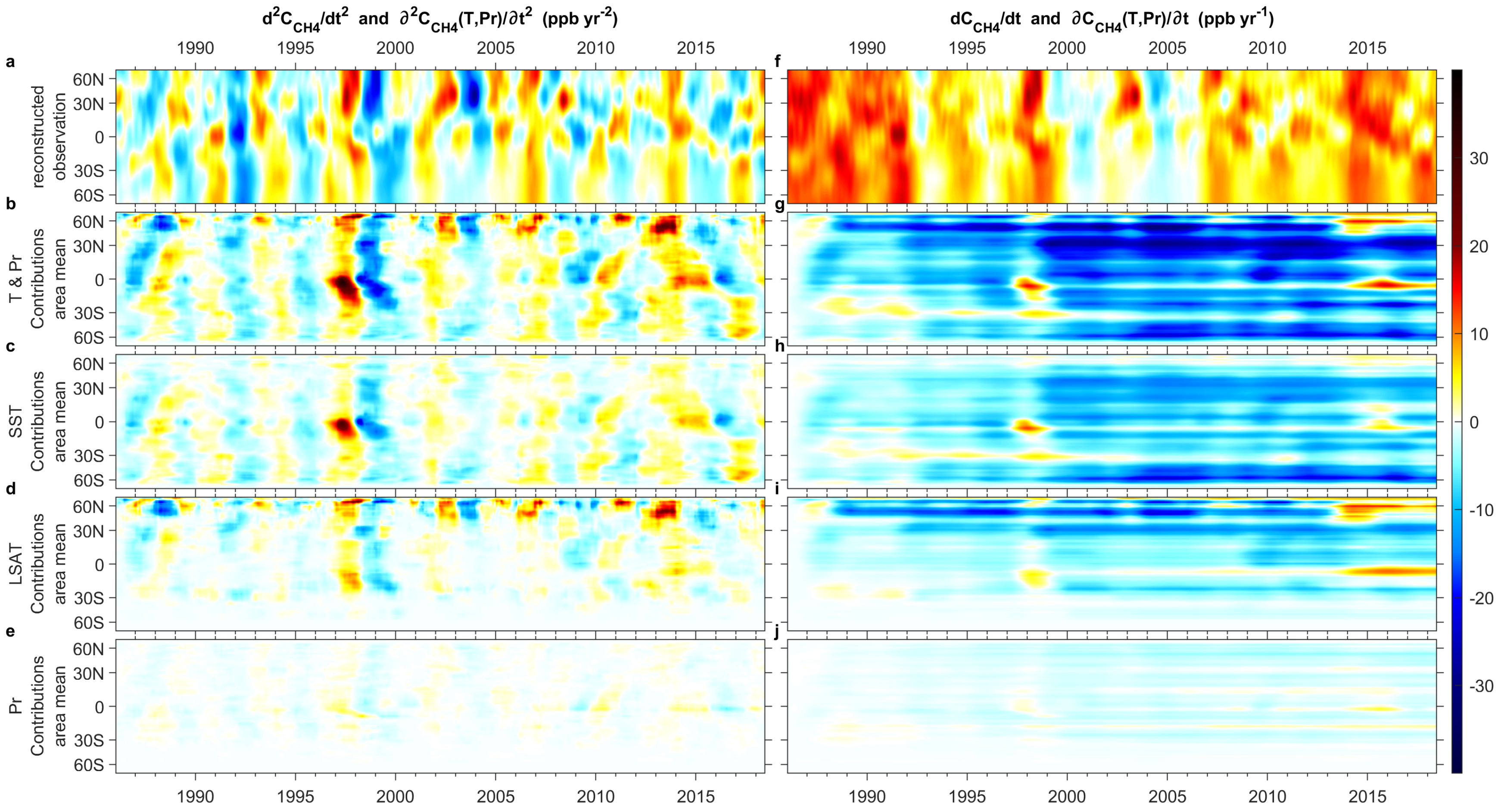

Supplementary Fig. 6. Comparison between the observed *d^2C_{CH4}/dt^2* (a), *dC_{CH4}/dt* (f), and estimated ∂^2C_{CH4}(*T&Pr*)*/*∂t^2 (b-e) and ∂C_{CH4}(*T&Pr*)*/*∂t (g-j) *c*-contributions given by *md₃nIF* and area-mean. The results are unable to properly reflect the interannual variability, especially it tends to underestimate the positive $\partial^2C_{CH4}(T\&Pr)/\partial t^2$ c-contributions and result in the net negative $\partial C_{CH4}(T\&Pr)/\partial t$ throughout most the times and latitudes.
